# Supplementary material for: Simulation and inference methods for non-Markovian stochastic reaction networks
Source: PLoS Comput Biol. 2026 Aug 26;22(8):e1014687. doi: 10.1371/journal.pcbi.1014687 (PMC13596995; doi:10.1371/journal.pcbi.1014687)
Supplement: Supplementary file 1 [file pcbi.1014687.s001.pdf]

Supporting information for "Simulation and inference methods for non-Markovian stochastic biochemical reaction networks"

Thomas P. Steele[1], David J. Warne[1,2,3*]

1 School of Mathematical Sciences, Queensland University of Technology, Brisbane, Queensland 4001, Australia
2 Centre for Data Science, Queensland University of Technology, Brisbane, Queensland 4001, Australia
3 ARC Centre of Excellence for Mathematical Analysis of Cellular Systems (MACSYS), Queensland University of Technology, Brisbane, Queensland 4001, Australia

* david.warne@qut.edu.au

S1 Appendix. Coupling and continuation probabilities

The theory of multifidelity ABC does not strictly require a coupling mechanism between the low-fidelity, $\tilde{y}_{\text{sim}}$, and high-fidelity simulators y_{sim}. However, to obtain meaningful performance improvements coupling schemes are extremely effective. In this appendix, we demonstrate the importance of coupling scheme as it relates to optimal configuration of the multifidelity continuation probability function.

We denote $\tilde{C}$ as the computational cost of generating a low-fidelity simulation and C as the computational cost of generating a high-fidelity simulation. Furthermore we assume $\tilde{C}/C \ll 1$. For a continuation probability function, $\mu(\tilde{y}_{\text{sim}})$, of the form given in Eq. (15) of the main manuscript, Prescott and Baker [1] show that the optimal continuation probabilities are

$$\mu_a^* = \left(\frac{\tilde{c}p_{\text{fp}}}{c_{\text{p}}(p_{\text{tp}} - p_{\text{fp}})}\right)^{1/2}, \text{ and } \mu_r^* = \left(\frac{\tilde{c}p_{\text{fn}}}{c_n(p_{\text{tp}} - p_{\text{fp}})}\right)^{1/2}, \tag{1}$$

where $\tilde{c} = \mathbb{E}[\tilde{C}]$, is the expected cost of the low-fidelity simulator, $c_{\text{p}} = \mathbb{E}[C \mid \rho(\tilde{y}_{\text{sim}}, y_{\text{obs}}) \leq \varepsilon]$ is the expected cost of continuing to a high-fidelity simulation following an accepted low-fidelity simulation, $c_{\text{n}} = \mathbb{E}[C \mid \rho(\tilde{y}_{\text{sim}}, y_{\text{obs}}) > \varepsilon]$ is the expected cost of continuing to a high-fidelity simulation following a rejected low-fidelity simulation, $p_{\text{fp}} = \Pr(\rho(y_{\text{sim}}, y_{\text{obs}}) > \varepsilon \mid \rho(\tilde{y}_{\text{sim}}, y_{\text{obs}}) \leq \varepsilon)$ is the probability of a false positive (accepted low-fidelity and rejected high-fidelity), $p_{\text{tp}} = \Pr(\rho(y_{\text{sim}}, y_{\text{obs}}) \leq \varepsilon \mid \rho(\tilde{y}_{\text{sim}}, y_{\text{obs}}) \leq \varepsilon)$ is the probability of a true positive (accepted low-fidelity and accepted high-fidelity), and $p_{\text{fn}} = \Pr(\rho(y_{\text{sim}}, y_{\text{obs}}) \leq \varepsilon \mid \rho(\tilde{y}_{\text{sim}}, y_{\text{obs}}) > \varepsilon)$ is the probability of a false negative (rejected low-fidelity and accepted high-fidelity).

The smaller μ_a^* and μ_r^* are, the less frequently high-fidelity simulations are needed to correct for errors accumulated though the use of the low-fidelity simulator, leading to computational gains. In the context of non-Markovian schemes we consider in this paper, we have no control over c_{p} and c_{n} as it is based on nM-NRM (Alg. 3), however, $\tilde{c}$ can be reduced by increasing the time step τ in the nM-TLM (Alg. 4). Doing so will increase the error rate in the low-fidelity accept/reject decision as a predictor for the high-fidelity accept/reject decision. That is, as τ increases, p_{fp} and p_{fn} will increase and p_{tp} will decrease, leading potentially only negligible reduction in μ_a^* and μ_r^* below unity.

When coupling is introduced using Algs. 5 and 6, the low-fidelity and high-fidelity simulations become positively correlated. This, in turn, leads to improved predictive characteristics with decreased p_{fp} and p_{fn} and increased p_{tp}. This effect is visualised in Fig. A for simulation pairs of the discrepancy metric under the low-fidelity and high-fidelity simulation.

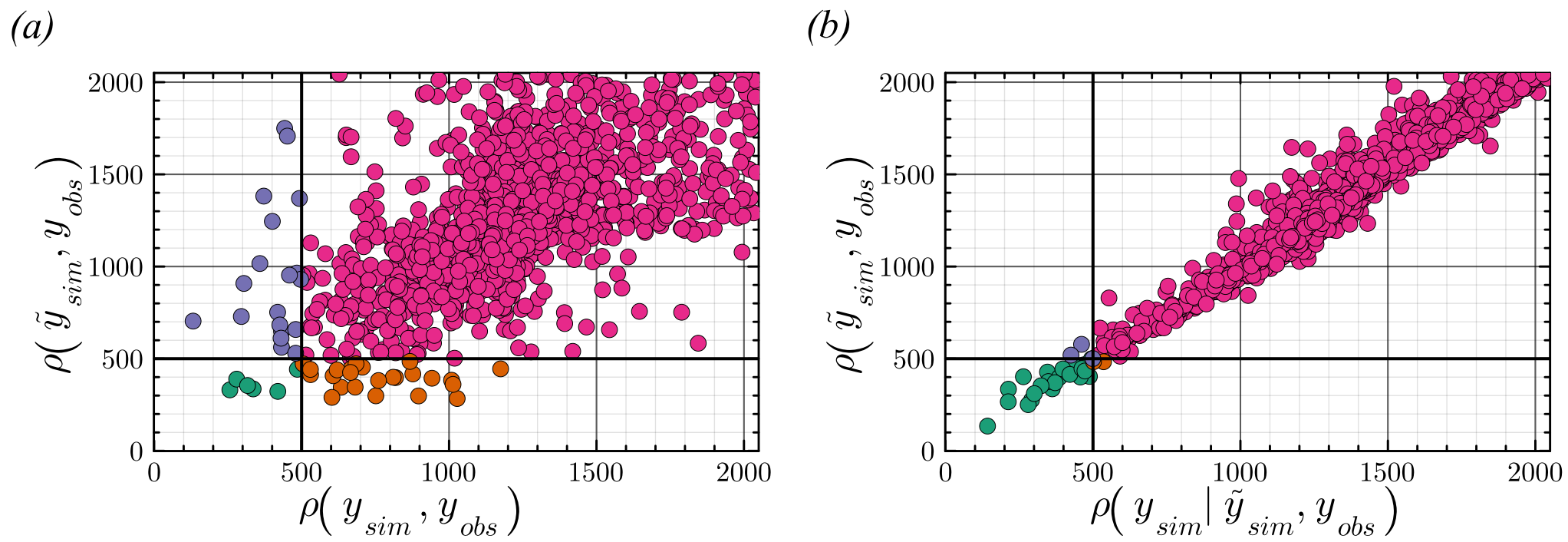

Fig. A. Scatter plots interpreted as confusion matrices of discrepancy measures from the observed data, for (a) uncoupled and (b) coupled sample paths using $\tau = 0.25$. The acceptance threshold of $\varepsilon = 500$ is indicated (sold black lines) to highlight the true positive region (green circles), true negative region (pink circles), false positive region (orange circles) and false negative region (purple circles).

References

1. Prescott TP, Baker RE. Multifidelity Approximate Bayesian Computation. SIAM/ASA Journal on Uncertainty Quantification. 2020 Jan;8(1):114-38. doi:10.1137/18m1229742.
