# Supplementary material for: Simulation and inference methods for non-Markovian stochastic reaction networks
Source: PLoS Comput Biol. 2026 Aug 26;22(8):e1014687. doi: 10.1371/journal.pcbi.1014687 (PMC13596995; doi:10.1371/journal.pcbi.1014687)
Supplement: Supplementary file 2 [file pcbi.1014687.s002.pdf]

Supporting information for “Simulation and inference methods for non-Markovian stochastic biochemical reaction networks”

Thomas P. Steele[1], David J. Warne[1,2,3*]

1 School of Mathematical Sciences, Queensland University of Technology, Brisbane, Queensland 4001, Australia
2 Centre for Data Science, Queensland University of Technology, Brisbane, Queensland 4001, Australia
3 ARC Centre of Excellence for Mathematical Analysis of Cellular Systems (MACSYS), Queensland University of Technology, Brisbane, Queensland 4001, Australia

* david.warne@qut.edu.au

S2 Appendix. Coupling exact and approximate paths

Here we develop a coupling scheme to generate sample path pairs $\{(\mathbf{X}(t), \mathbf{Z}(t))\}_{t\geq t_0}$ such that the sample paths are positively correlated for any $t > t_0$. This enables computation cost reductions using MLMC or multifidelity schemes. Our approach involves two steps: (i) we perform the completion of Poisson processes using firing counts and interval lengths from a realisation of $\mathbf{Z}(t)$ using nM-TLM, then (ii) we decompose the delayed reaction channels in the nM-TLM to match the exact setting in nM-NRM where copy numbers for all delayed chemical species are at most unity.

Suppose we simulate $\mathbf{Z}(t)$ for n time steps of length τ with $t_i = t_0 + i\tau$ for $i = 0, 1, 2, \ldots, n$. At time t_i for a given reaction channel $j \in \mathcal{R}$ (resp. $\kappa \in \mathcal{D}_j$ and $j \in \mathcal{R}_D$ for progressing delay channels), denote $P_{i,j}$ (resp. $P_{i,j}^{\kappa}$) to be the number of events that occur over the time interval $[t_i, t_{i+1}) = [t_i, t_i + \tau)$. We can relate this time interval to equivalent intervals for the unit rate processes, $Y_j(\cdot)$ (resp. $Y_j^{\kappa}(\cdot)$), via the random time-change formulae. That is the interval, $[t_{i,j,u}, t_{i+1,j,u})$ (resp. $[t_{i,j,u}^{\kappa}, t_{i+1,j,u}^{\kappa})$), over which the unit rate process fires $P_{i,j}$ (resp. $P_{i,j}^{\kappa}$) times, is given by,

$$t_{i,j,u} = \Lambda_j(t_i; \mathbf{Z}(t_0), t_0), \text{ and } t_{i+1,j,u} = \Lambda_j(t_{i+1}; \mathbf{Z}(t_0), t_0) = t_{i,j,u} + \Lambda_j(\tau; \mathbf{Z}(t_i), t_i), \quad (1)$$

and respectively,

$$t_{i,j,u}^{\kappa} = \Lambda_j^{*}(t_i; \mathbf{Z}(t_0), t_0, t_{j,0}^{*,\kappa}), \text{ and } t_{i+1,j,u}^{\kappa} = \Lambda_j^{*}(t_{i+1}; \mathbf{Z}(t_0), t_0, t_{j,0}^{*;\kappa}) = t_{i,j,u}^{\kappa} + \Lambda_j^{*}(\tau; \mathbf{Z}(t_i), t_i, t_{j,0}^{*;\kappa}). \quad (2)$$

For a homogeneous Poisson processes, $Y_j(\cdot)$ (resp. $Y_j^{\kappa}(\cdot)$), the time of an event that occurs within the interval $[t_{i,j,u}, t_{i+1,j,u})$ (resp. $[t_{i,j,u}^{\kappa}, t_{i+1,j,u}^{\kappa})$) is a uniformly distributed random variable over the interval. This leads to Alg. 1 for constructing a sequence of event times for the unit rate Poisson processes based on the approximate sample path generated through nM-TLM. The resulting sets of event times $\mathcal{T}_j$ for $j \in \mathcal{R}$ can be used in place of sampling the next reaction intervals $r_{j,u} \sim \text{Exp}(1)$ in the nM-NRM, that is the mth sample of $r_{j,u}$ can be computed using $r_{j,u}^{(m)} = t_{j,u}^{(m)} - t_{j,u}^{(m-1)}$ where $t_{j,u}^{(m)} = \min\left(\mathcal{T}_j \backslash \{t_{j,u}^{(1)}, t_{j,u}^{(2)}, \ldots, t_{j,u}^{(m-1)}\}\right)$.

Algorithm 1 Poisson process completion

Require: Event counts, $P_{i,j}$, for $j \in \mathcal{R}$, and $P_{i,j}^{\kappa}$ for $\kappa = 1, 2, \ldots, K_j$ where $K_j = \sum_{i=1}^{n} \mathbb{1}_{[1,\infty)}(P_{i,j})$ and $j \in \mathcal{R}_D$ from a realisation of Alg. **??** for times $t_i = t_0 + i\tau$ with $i = 0, 1, \ldots, n,$;

Set $\mathcal{T}_j = \{0\}$ as the set of event times for $Y_j(\cdot)$ for $j \in \mathcal{R}$;

Set $\mathcal{T}_j^{\kappa} = \{0\}$ as the set of event times for $Y_j^{\kappa}(\cdot)$ for $\kappa = 1, 2, \ldots, K_j$ and $j \in \mathcal{R}_D$;

for $j \in \mathcal{R}$ **do**

  for $i = 1, ..., n$ **do**

    Compute interval for unit rate process $[t_{i,j,u}, t_{i+1,j,u})$ using Eq. (1)

    Sample event times $t_{j,u}^{(m)} \sim \text{Uniform}(t_{i,j,u}, t_{i+1,j,u})$ for $m = 1, 2, \ldots, P_{i,j}$;

    $\mathcal{T}_j \leftarrow \mathcal{T}_j \cup \{t_{j,u}^{(1)}, t_{j,u}^{(2)}, \ldots, t_{j,u}^{(m)}\}$

    if $j \in \mathcal{R}_D$ **then**

      for $\kappa = 1, 2, \ldots, K_j$ **do**

        Compute interval for unit rate process $[t_{i,j,u}^{\kappa}, t_{i+1,j,u}^{\kappa})$ using Eq. (2)

        Sample event times $t_{j,u}^{\kappa,(m)} \sim \text{Uniform}(t_{i,j,u}^{\kappa}, t_{i+1,j,u}^{\kappa})$ for $m = 1, 2, \ldots, P_{i,j}^{\kappa}$;

        $\mathcal{T}_j^{\kappa} \leftarrow \mathcal{T}_j^{\kappa} \cup \{t_{j,u}^{\kappa,(1)}, t_{j,u}^{\kappa,(2)}, \ldots, t_{j,u}^{\kappa,(m)}\}$

      end for

    end if

  end for

end for

Alg. 1 is almost all that is needed to implement the nM-NRM using the event times $\mathcal{T}_j$ (resp. $\mathcal{T}_j^{\kappa}$) for the unit time processes $Y_j(\cdot)$ (resp. $Y_j^{\kappa}(\cdot)$). However the process $Y_j^{\kappa}(\cdot)$ constructed here in Alg. 1 is not identical to the $Y_j^{k}(\cdot)$ in the nM-NRM. This is due to the fact that within the nM-TLM groups of delayed chemical species are handled with a single delayed reaction channel. In contrast, nM-NRM handles each initiated delayed reaction with a unique delay chemical species having its own reaction channel. This requires the groups to be disassembled into distinct reaction channels with a single event time.

Following from Eq. (1) for a delayed channel $j \in \mathcal{R}_D$, the set $\mathcal{T}_j$ contains all initiation times, and the sequence of sets $\mathcal{T}_j^1, \mathcal{T}_j^2, \ldots \mathcal{T}_j^{K_j}$ contains all the completion times. The group disassembly proceeds as follows, the first $|\mathcal{T}_j^1|$ initiation events from $\mathcal{T}_j$ will be assigned a completion times from $\mathcal{T}_j^1$ at random uniformly without replacement until $\mathcal{T}_j^1 = \emptyset$, then the next $|\mathcal{T}_j^2|$ initiation events from $\mathcal{T}_j$ will be assigned a completion times from $\mathcal{T}_j^2$ at random uniformly without replacement until $\mathcal{T}_j^2 = \emptyset$, and so on until either $\mathcal{T}_j = \emptyset$ or $\mathcal{T}_j^{K_j} = \emptyset$. Combining the completion of the Poisson process with this disassembly procedure provides a set of event times for the unit time Poisson processes needed for the coupled nM-NRM. This effectively proceeds via implementing nM-NRM with next event times being determined via the Alg. 1. The explicit implementation of this coupled nM-NRM is provided in . Importantly, the process generates an exact realisation from the non-Markovian biochemical reaction network that is correlated to a realisation of the nM-TLM approximation through the use of the same Poisson process clocks.

Using the complete Poisson processes, we arrive at the coupled non-Markovian next reaction method (C-nM-NRM). The algorithm generates an exact realisation of a non-Markovian reaction network that is correlated to given τ-leaping realisation as generated from Alg. 4. As discussed in the main text, the method presented here in Alg. 2 is effectively the nM-NRM that is driven by the completed unit-time Poisson processes.

Algorithm 2 Coupled non-Markovian Next Reaction Method (C-nM-NRM)

Require: A non-Markovian biochemical reaction network with $\mathcal{N}$ species and $\mathcal{M}$ reaction channels, of which $\mathcal{D}$ are delay reactions (Eq. (6)); an initial time $t_0 \leq 0$; a final time $T > t_0$; an initial system state $\mathbf{x}_0 = \mathbf{X}(t_0)$; event time sets $\mathcal{T}_j$ for $j \in \mathcal{R}$ and $\mathcal{T}_j^{\kappa}$ for $\kappa = 1, 2, \ldots, K_j$ and $j \in \mathcal{R}_D$ as constructed from Alg. 5;

Initialise state $\mathbf{X} \leftarrow \mathbf{x}_0$, time $t \leftarrow t_0$, internal times $t_j \leftarrow 0$ for $j \in \mathcal{R}$, first reaction intervals, $r_{j,u} \leftarrow \min(\mathcal{T}_j)$ for $j \in \mathcal{R}$, and delay sets $\mathcal{D}_j \leftarrow \emptyset$, $j \in \mathcal{R}_D$; and set event counters $m_j = 1$.

while $t < T$ **do**

  Update next reaction times τ_j for $j \in \mathcal{R}_I$, and τ_j^k for $k \in \mathcal{D}_j$ and $j \in \mathcal{R}_D$ (Eqs. (22)–(23));

  Find $\tau_{\mu_1} \leftarrow \min_{j \in \mathcal{R}}(\tau_j)$, and $\tau_{\mu_2}^k \leftarrow \min_{j \in \mathcal{R}_D, k \in \mathcal{D}_j}(\tau_j^k)$;

  Set next event time $\tau_{\min} \leftarrow \min(\tau_{\mu_1}, \tau_{\mu_2}^k)$

  Update remaining intervals $r_{j,u}$ for $j \in \mathcal{R}$, and $r_{j,u}^k$ for $k \in \mathcal{D}_j$ $j \in \mathcal{R}_D$ (Eqs (20)–(21));

  Update internal times $t_j \leftarrow t_j + \tau_{\min}$ for $j \in \mathcal{R}$, and $t_j^k \leftarrow t_j^k + \tau_{\min}$ for $k \in \mathcal{D}_j$ $j \in \mathcal{R}_D$;

  if $\tau_{\min} = \tau_{\mu_1}$ **then**

    if $m_j < |\mathcal{T}_j|$ **then**

      Set $m_j \leftarrow m_j + 1$;

      Set $t_{j,u}^{(m_j)} \leftarrow \min(\mathcal{T}_j \backslash \{t_{j,u}^{(1)}, t_{j,u}^{(2)}, \ldots, t_{j,u}^{(m_j-1)}\})$ and $t_{j,u}^{(m_j)} \leftarrow \min(\mathcal{T}_j \backslash \{t_{j,u}^{(1)}, t_{j,u}^{(2)}, \ldots, t_{j,u}^{(m_j-2)}\})$;

      Set $r_{\mu_1,u} \leftarrow t_{j,u}^{(m_j)} - t_{j,u}^{(m_j-1)}$;

    else

      Set $r_{\mu_1,u} \sim \text{Exp}(1)$;

    end if

    if $\mu_1 \in \mathcal{R}_I$ **then**

      Set $\nu \leftarrow \nu_{*,\mu_1}$;

    else

      Set $\nu \leftarrow \eta_{\mu_1} - \nu_{*,\mu_1}^-$;

      Set $t_{\mu_1,0}^{*,k'} \leftarrow t + \tau_{\mu_1}$, and $t_{\mu_1}^{k'} \leftarrow 0$;

      Set $k' \leftarrow X_{\mu_1}^* + 1$;

      for $\kappa \in [1, 2, \ldots, K_j]$ **do**

        if $\mathcal{T}_j^{\kappa} \neq \emptyset$ **then**

          Select $t_{j,u}^{\kappa,(m)}$ from $\mathcal{T}_j^{\kappa}$ with probability $1/|\mathcal{T}_j^{\kappa}|$;

          Set $r_{\mu_1,u}^{k'} \leftarrow t_{j,u}^{\kappa,(m)} - t_{\mu_1,0}^{*,k'}$ and $\mathcal{T}_j^{\kappa} \leftarrow \mathcal{T}_j^{\kappa} \backslash \{t_{j,u}^{\kappa,(m)}\}$;

          Break loop;

        end if

      end for

      if $\kappa > K_j$ **then**

        Set $r_{\mu_1,u}^{k'} \sim \text{Exp}(1)$

      end if

      Initiate delayed reaction $\mathcal{D}_{\mu_1} \leftarrow \mathcal{D}_{\mu_1} \cup \{k'\}$;

    end if

  else

    Set $\nu \leftarrow \nu_{*,\mu_2}^+ - \eta_{\mu_2}$;

    Complete delayed reaction $\mathcal{D}_{\mu_2} \leftarrow \mathcal{D}_{\mu_2} \backslash \{k\}$;

  end if

  Update the system state $\mathbf{X} \leftarrow \mathbf{X} + \nu$, and time $t \leftarrow t + \tau_{\min}$;

end while
