# Supplementary material for: Simulation and inference methods for non-Markovian stochastic reaction networks
Source: PLoS Comput Biol. 2026 Aug 26;22(8):e1014687. doi: 10.1371/journal.pcbi.1014687 (PMC13596995; doi:10.1371/journal.pcbi.1014687)
Supplement: Supplementary file 3 [file pcbi.1014687.s003.pdf]

Supporting information for "Simulation and inference methods for non-Markovian stochastic biochemical reaction networks"

Thomas P. Steele[1], David J. Warne[1,2,3*]

1 School of Mathematical Sciences, Queensland University of Technology, Brisbane, Queensland 4001, Australia
2 Centre for Data Science, Queensland University of Technology, Brisbane, Queensland 4001, Australia
3 ARC Centre of Excellence for Mathematical Analysis of Cellular Systems (MACSYS), Queensland University of Technology, Brisbane, Queensland 4001, Australia

* david.warne@qut.edu.au

S3 Appendix. Inter-event time distribution

To characterise the stochastic dynamics introduced by delay reactions, we begin by deriving the inter-event time distribution associated with their conclusion events. This leads naturally to a view of the system as a non-Markovian process, and motivates a generalisation of the reaction channel formalism central to stochastic simulation.

At time $t > t_0 > 0$ consider a delayed chemical species $\mathcal{X}_j^{*,k}$, created at initiation time $t_{j,0}^{*,k} < t$ and whose completion time is a random variable $T > t$. Suppose the reaction completes with propensity $\lambda_j^*(\mathbf{X}(t), t_j^k)$, where $\mathbf{X}(t)$ is the system state at time t and $t_j^k = t - t_{j,0}^{*,k}$ is the internal time since the reaction initiation. Then, the conditional probability that the reaction will complete in the interval $[t, t + \Delta t)$ is given by,

$$\Pr(T < t + \Delta t \mid T > t) = \lambda_j^*(\mathbf{X}(t + \Delta t), t_j^k + \Delta t)\Delta t + \mathcal{O}\left(\Delta t^2\right).$$

Let $g_k(\tau) = \Pr(T > t + \tau \mid T > t)$ denote the survival probability after some time τ. Then, over an interval $\tau + \Delta t$ we have,

$$g_k(\tau + \Delta\tau) = g_k(\tau)\left(1 - \lambda_j^*\left(\mathbf{X}(t + \tau + \Delta t), t_j^k + \tau + \Delta t\right)\Delta t + \mathcal{O}\left(\Delta t^2\right)\right). \quad (1)$$

Rearranging Eq. (1) and taking the limit $\Delta t \to 0$ gives an ODE for the survival function,

$$\frac{\mathrm{d}g_k(\tau)}{\mathrm{d}\tau} = -g_k(\tau)\lambda_j^*(\mathbf{X}(t + \tau), t_j^k + \tau), \qquad g_k(0) = 1 \quad (2)$$

Here, Eq. (2) admits the solution,

$$g_k(\tau) = \exp\left(-\int_0^\tau \lambda_j^*\left(\mathbf{X}(t + s), t_j^k + s\right)\,\mathrm{d}s\right). \quad (3)$$

The solution to the survival function (Eq. (3)) mirrors the arrival time distribution of an inhomogeneous Poisson process with a propensity depending on internal time t_k. This leads to the system as a whole being non-Markovian due to the internal time dependence on the survival time, and the dependence on the history since the internal time depends on the initiation time $t_0^{*,k}$. We note that our framework is applicable for all valid propensity functions, that is, $\lambda(\mathbf{X}, t) \geq 0$ for all $X \in \mathbb{N}^{\mathcal{N}+\mathcal{D}}$ and $t > 0$, and $\lim_{t\to\infty}\int_0^t \lambda(\mathbf{X}, u)\,\mathrm{d}u = \infty$. This admits a wide class of possible waiting time distributions including, uniform, heavy-tailed distributions such as half-Cauchy, and deterministic delays via piecewise-constructed hazard functions.
